# Integration of scRNA-Seq and TCGA RNA-Seq to Analyze the Heterogeneity of HPV+ and HPV- Cervical Cancer Immune Cells and Establish Molecular Risk Models

**DOI:** 10.3389/fonc.2022.860900

**Published:** 2022-06-01

**Authors:** Erdong Wei, Amin Reisinger, Jiahua Li, Lars E. French, Benjamin Clanner-Engelshofen, Markus Reinholz

**Affiliations:** ^1^ Department of Dermatology and Allergy, Ludwig-Maximilians-Universität München (LMU) Munich, University Hospital, Munich, Germany; ^2^ Dr. Phillip Frost Department of Dermatology & Cutaneous Surgery , Miller School of Medicine, University of Miami, Miami, FL, United States

**Keywords:** cervical cancer, HPV - human papillomavirus, scRNA seq, immune cells (ICs), gene signature, prognosis, carcinoma

## Abstract

**Background:**

Numerous studies support that Human papillomavirus (HPV) can cause cervical cancer. However, few studies have surveyed the heterogeneity of HPV infected or uninfected (HPV+ and HPV-) cervical cancer (CESC) patients. Integration of scRNA-seq and TCGA data to analyze the heterogeneity of HPV+ and HPV- cervical cancer patients on a single-cell level could improve understanding of the cellular mechanisms during HPV-induced cervical cancer.

**Methods:**

CESC scRNA-seq data obtained from the Gene Expression Omnibus (GEO) database and the Seurat, Monocle3 package were used for scRNA-seq data analysis. The ESTIMATE package was used for single-sample gene immune score, CIBERSORT package was used to identify immune scores of cells, and the “WGCNA” package for the weighted correlation network analysis. Univariate Cox and LASSO regression were performed to establish survival and relapse signatures. KEGG and GO analyses were performed for the signature gene. Gene Expression Profiling Interactive Analysis was used for Pan-cancer analysis.

**Results:**

In the HPV+ CESC group, CD8+ T cells and B cells were down-regulated, whereas T reg cells, CD4+ T cells, and epithelial cells were up-regulated according to scRNA-seq data. Survival analysis of TCGA-CESC revealed that increased expression of naive B cells or CD8+ T cells favors the survival probability of CESC patients. WGCNA, univariate Cox, and LASSO Cox regression established a 9-genes survival signature and a 7-gene relapse model. Pan-cancer analysis identified IKZF3, FOXP3, and JAK3 had a similar distribution and effects in HPV-associated HNSC.

**Conclusion:**

Analysis of scRNA-seq and bulk RNA-seq of HPV+ and HPV- CESC samples revealed heterogeneity from transcriptional state to immune infiltration. Survival and relapse models were adjusted according to the heterogeneity of HPV+ and HPV- CESC immune cells to assess the prognostic risk accurately. Hub genes represent similar protection in HPV- associated HNSC while showing irrelevant to other potential HPV-related cancers.

## Introduction

In 2020, the World Health Organization (WHO) reported 604,127 new cases of cervical cancer (CESC) and 341,831 CESC-related casualties (1). Seventy percent of patients of the CESC and CESC-related deaths were Human papillomavirus (HPV) type 16 and 18 positive (2). The HPV, a non-enveloped double-stranded DNA virus, is a well-studied transmitter of sexual infections worldwide (1). Many HPV infections have no symptoms, and 90% resolve spontaneously within two years (2). However, in some cases (3), HPV infections could result in warts or precancerous lesions, which increase the risk of cancer, including vulva, vagina, penis, and anus (4).

Single-cell RNA sequencing (scRNA-seq) is a technique used to define the global gene expression profile of a single cell utilizing optimized next-generation sequencing. This allows the exploration of previously hidden heterogeneity in a cell population (5). In previous studies, scRNA-seq was the technique of choice for investigating cancer and adjacent normal tissues. In Li`s study (6), scRNA-seq was performed to compare the expression differences between CESC-derived endothelial cells and normal endothelial cells revealing marker genes associated with cancer endothelial cells. In comparison with scRNA-seq, the advantage of The Cancer Genome Atlas (TCGA) data lies within the ability to integrate RNA samples and clinical information on a larger scale, which enables consecutive analysis of the clinical significance of genes. Bezzecchi et al. used TCGA RNA-seq to analyze the HPV+ and HPV- Neck squamous cell carcinoma (HNSC) revealing that the overexpression of histone fold domain subunits and NF-YAs is counteracting cancer progression in HPV+ patients (7).

We hypothesized that HPV has an effect at multiple levels. Hence, we aim to reveal the heterogeneity from transcriptional to immune infiltration between HPV+ and HPV- samples by scRNA-seq as well as bulk RNA sequencing. Then, we try to link the survival and relapse with genes based on the difference between HPV+ and HPV- samples. Further, we analyzed the role of hub genes in other potential HPV- associated cancers to know whether HPV infections have a consistent effect on different cancers.

## Methods

### Data Collection and Processing

Single-cell RNA sequencing data of cervical cancer with HPV+ and HPV- (GSM5236544, GSM5236545, GSM5236546, GSM5236547) were downloaded from Gene Expression Omnibus (GEO) database (http://www.ncbi.nlm.nih.gov/geo/). Single-cell RNA sequencing data of HNSCC with HPV+ and HPV- (GSE139324) were downloaded from Gene Expression Omnibus (GEO) database Transcriptome profiling data and the corresponding clinical data were collected from GSE GEO database (GSE142583, GSE6791, GSE181805, GSE190224) and TCGA-CESC *via* the Sangerbox tools, a free online platform for data analysis (http://www.sangerbox.com/tool).

### Single-Cell RNA Processing

The Seurat package was used to explore the transcriptional heterogeneity of cells within the HPV+ and HPV- tumor microenvironment for integration, pre-process, batch effect reduction, and non-linear dimension reduction of the datasets (8). The FindCluster () function was used to cluster cells. The Findmarkers () function was used to get marker genes in the clusters. SingleR was used to annotate cells automatically. The pseudotime trajectory analysis was performed using the Monocle3 package with default settings (9).

### Immune Estimate

The immune score was estimated using ESTIMATE (Estimation of Stromal and Immune cells in Malignant Tumors using Expression data) (10). Immune cell proportions in the HPV+ and HPV- samples were analyzed utilizing cell type identification by calculating relative subsets of RNA transcripts (CIBERSORT) (11).

### DeRNA and WGCNA

Differentially expressed RNA (DeRNA) among HPV+ and HPV- groups were screened out through Linear Models for Microarray Analysis (limma) package in R software (12). The cutoff values of screening were |fold change (FC)| >1.2 and p < 0.05. The “WGCNA” package was used for the weighted correlation network analysis (WGCNA) (13).

### Molecular Risk Model Construction

The survival package’s coxph () function was used to fit the Cox risk regression, and a p-value of 0.05 was considered survival/relapse related (14). The least absolute shrinkage and selection operator (LASSO) method is a compression estimation that creates a more refined model by generating a penalty function, compressing certain coefficients while simultaneously setting some values to zero. As a result, the benefit of subset shrinking is preserved. A biased estimation for multicollinear data processing allows for selecting variables when estimating parameters, allowing for a better solution to the multicollinearity problem in regression analysis. We utilized the glmnet package for analysis, and for model construction, we used 10-fold cross-validation and LASSO Cox regression (15).

### Functional Enrichment Analysis

Gene Ontology (GO) and Kyoto Encyclopedia of Genes and Genomes (KEGG) enrichment analyses were performed to identify biological processes and pathways that were most related to hub genes with p < 0.05 as cutoff value (http://www.sangerbox.com/tool).

### HPV-Associated Pan Cancer Analysis

Gene Expression Profiling Interactive Analysis 2021 (GEPIA2021), a web-based tool, analyzed the hub genes expressions and overall survival in HPV-associated cancers (16). ScRNA-seq and RNA expression profiling of HPV+ and HPV- HNSCC were analyzed like 2.1 and 2.4.

## Result

### Data Integration and scRNA-Seq Data Analysis

Patients’ information was summarized from the corresponding clinical data in 46 samples with known HPV states (**Table 1**). Overall, the CESC patients in the HPV- group tended to be younger and more likely to have a lower tumor grade, but this is not significant (P=0.81) due to a massive number of high-grade patients in TCGA-CESC were excluded with incomplete HPV state.

**Table 1:** HPV+ and HPV- CESC patient information. The tumor stages and age information of HPV+ and HPV- CESC patients.

	HPV+ (N = 42)	HPV-(N = 4)	Total (N = 46)
**Tumor Stage**			
I	32 (69.57%)	3 (6.52%)	35 (76.09%)
II	5 (10.87%)	1 (2.17%)	6 (13.04%)
III	4 (8.70%)	0 (0.0e+0%)	4 (8.70%)
IV	1 (2.17%)	0 (0.0e+0%)	1 (2.17%)
**Age**			
Mean±SD	42.75 ± 11.47	37.00 ± 9.90	42.48 ± 11.36

**Figure 1:**
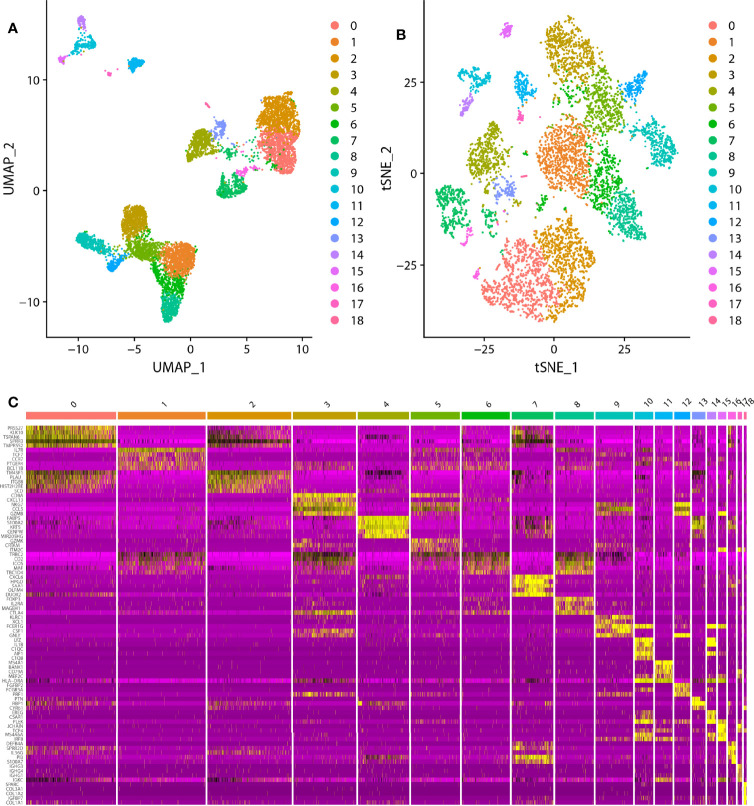
The dimension reduction of CESC scRNA-seq. **(A)** Separate clusters according to UAMP. **(B)** Separate clusters according to tSNE. **(C)** Top five marker genes of each cluster.

**Figure 2:**
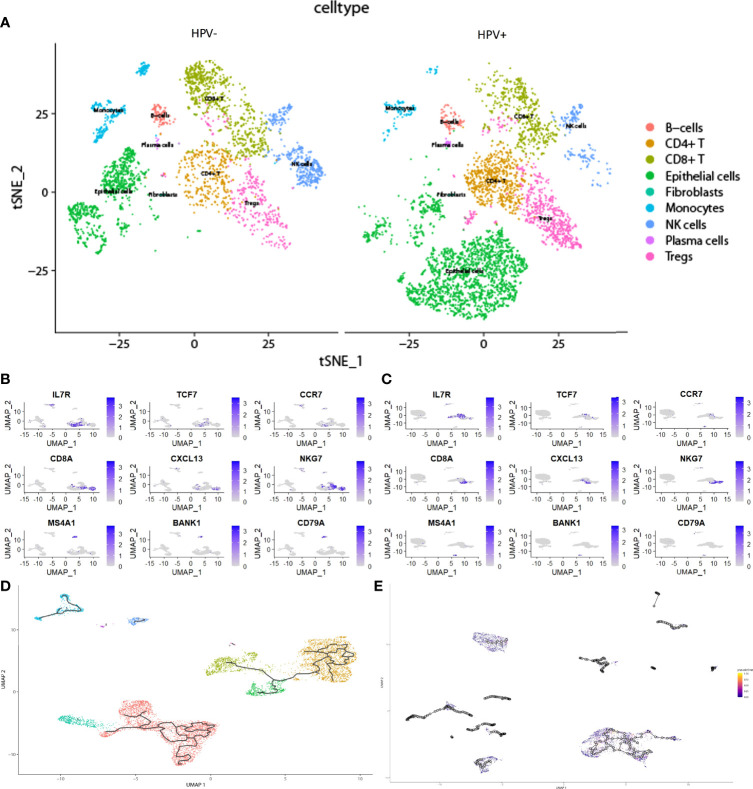
The heterogeneity in HPV+ and HPV- CESC scRNA-seq. **(A)** Cell type annotation of HPV+ and HPV- group. **(B)** Top marker genes of CD8+T, CD4+T, and B cells in HPV- group. **(C)** Top marker genes of CD8+T, CD4+T, and B cells in HPV+ group. **(D)** Pseudotime and trajectory analysis of HPV- group. **(E)** Pseudotime and trajectory analysis of HPV+ group.

In the scRNA analysis, 19 clusters were obtained by their gene expression patterns (11,899 marker genes) (**Figures 1A–C**). The annotation of cells by SingleR (**Figure 2A**) resulted in nine main immune cell type clusters based on their expression of immune cell markers. Based on the different abundance of marker genes expression in scRNA sequencing (**Figures 2B, C**), the dimension by UMAP and tSNE revealed reduced CD8^+^ T cell and B cell clusters, while an increase in Treg -, CD4^+^ T -, and epithelial cell clusters was observed within HPV+ CESC patients. Pseudotime trajectory analysis highlighted the heterogeneity of immune cell development between HPV+ and HPV- CESC patients (**Figures 2D, E**).

### Immune Cell Analysis

**Figure 3:**
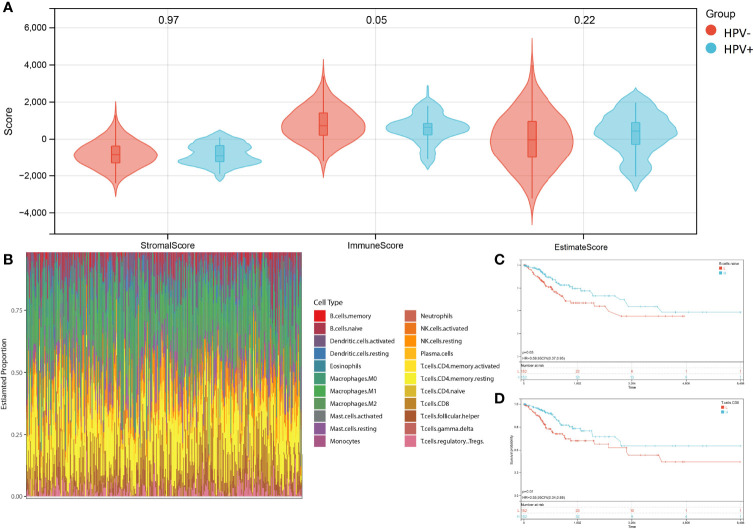
Character of immune infiltration. **(A)** Stromal score, Immune Score and Estimate Score of HPV+ AND HPV- group. **(B)** Bar charts of 22 immune cell proportions in CESC TCGA RNA-seq. **(C)** KM curves of different expression Naive B cell. **(D)** KM curves of different expression CD8+ T cell.

The advantage of large-scale integration of paired clinical information in TCGA-CESE transcriptional RNA data allowed the analysis of immune signatures between HPV+ and HPV- CESC groups by using the ESTIMATE algorithm with TCGA RNA data input (**Figure 3A**). CIBERSORT enabled the estimation of the CESC patients’ immune cell status (22 immune cell types) based on TCGA-CESE RNA count data (**Figure 3B**).

The performance of a survival analysis considering the immune status of the CESC patients revealed a low survival probability for patients with a smaller abundance of CD8+ T cells and naive B cells (**Figures 3C, D**). CD8+ T cells and naive B cells immune clusters were chosen as targets for subsequent analysis. Other immune cell clusters were excluded from subsequent analysis due to either not having significant expression between HPV+ and HPV- group or showed no statistical influence on the survival probability of CESC patients (**Supplementary Figure 1**). Genes in the cluster of epithelial cells were also excluded due to not being represented within the immune cell status (22 immune cells).

### Differentially Expressed RNA and WGCNA Analysis

**Figure 4:**
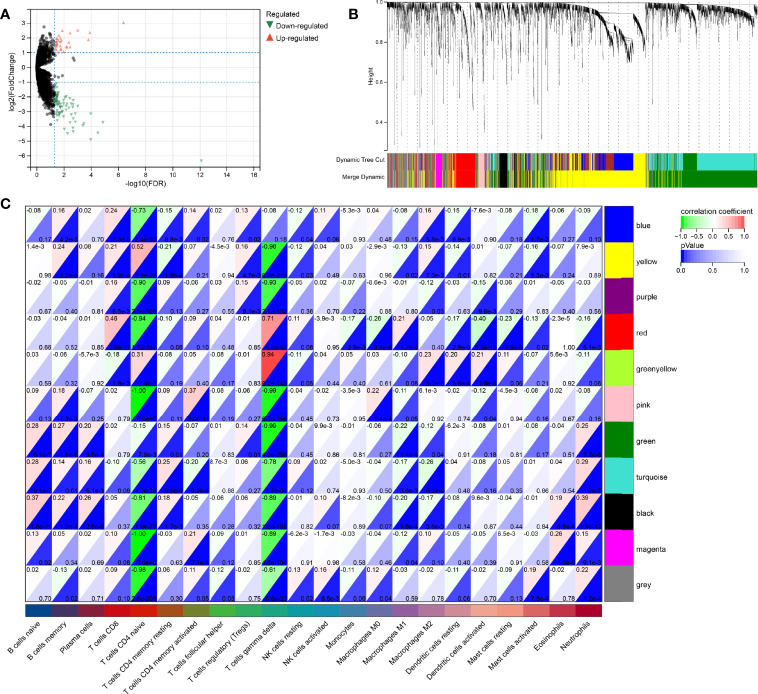
WGCNA analysis for DeRNA. **(A)** Volcano plot of DeRNA. **(B)** Clustering results of minded modules in DeRNA dataset. **(C)** The correlation between different modules and the proportions of 22 immune cells.

Since both the HPV+ and the HPV- group consists of CESC patients, the identification of DERNAs is aggravated. In contrast, the comparison of cancer and non-cancer groups allows a much simpler statistical analysis. To access the DERNAs between the HPV+ and HPV- CESC patients, an adjusted fold-change (1.2-fold change) and a standard p-value (<0.05) were set (**Figure 4A**) resulting in the identification of 6317 DERNAs. After creating a matrix containing the identified DERNAs, a WGCNA analysis (soft threshold = 5) was performed. The construction of a scale-free co-expression network enabled the identification of gene features related to CD8+ T cells and naive B cells. In total, 11 modules were generated (**Figure 4B**), of which the red had the highest correlation with the CD8+T score (r=0.46, P=2.8e−17, **Figure 4C**), and the black had the highest correlation with the naive B cells score (r=0.37, P=1.8e−11, **Figure 4C**). The results show that the important elements of the red module and black module represent CD8+ T cells and naive B cells respectively, finally obtaining 249 hub genes (MM>0.8 and GS>0.1 from the two modules.

### Genetic Risk Model

**Figure 5:**
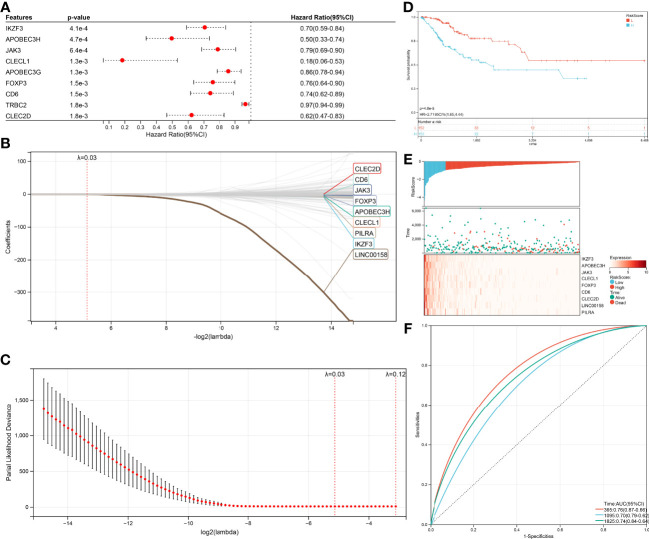
Construction of the Cox and LASSO Cox model. **(A)** Top nine survival-related genes in Cox model. **(B)** The trajectory of each independent variable in the survival-related model: the horizontal axis represents the log2 value of the independent variable lambda, and the vertical axis represents the coefficient of the independent variable. **(C)** The confidence interval under each lambda in the survival-related model. **(D)** Top9 relapse-related genes in Cox model. **(E)** The trajectory of each independent variable in the relapse-related model: the horizontal axis represents the log2 value of the independent variable lambda, and the vertical axis represents the coefficient of the independent variable. **(F)** The confidence interval under each lambda in the relapse-related model.

Univariate Cox (proportional hazards model) proportional risk models were taken to analyze the hub genes, which related to CD8+ T cells, Naive B cells, and differently expressed between HPV+ and HPV- CESC. Two hundred genes were significantly associated with overall survival (log-rank test p<0.05, **Figure 5A**). In the survival univariate Cox proportional risk models, 39 genes (HR>1) were recognized as increased in hazard, 161 genes (HR<1) were recognized as reduction in hazard. Then, LASSO regression was used to solve the multicollinearity problem and minimize the number of genes in the risk model, as shown in **Figure 5B** LASSO Cox regression analysis, and each independent variable’s change trajectory. Next, internal 10-fold cross-validation was performed after LASSO-cox regression to get a superior model (**Figure 5C**), When lambda = 0.0285755570440733, the model is optimum, and nine genes (IKZF3, APOBEC3H, JAK3, CLECL1, FOXP3, CD6, CLEC2D, LINC00158, PILRA) were chosen to build a survival risk model. The final 9-gene signature is as follows:

*RiskScore* = –0.110045311944626 ∗ *IKZF*3 – 0.180200464406653 ∗ *APOBEC*3*H* – 0.009738330408125074 ∗ *JAK*3 – 0.100574502555081 ∗ *CLECL*1 – 0.0109656212011594 ∗*FOXP*3 – 0.00569462037861635 ∗ *CD*6 – 0.00590561916507251 ∗ *CLEC*2*D* – 0.279175668959309 ∗ *LINC*00158 – 0.00677990623712316 ∗ *PILRA*

Due to nine genes in the survival risk model having a negative coefficient, these genes could be considered as favorable, the higher these genes expression, the lower RiskScore will be calculated. The TCGA-CESC samples with corresponding survival state and survival time were classified as high or low risk with median standardization. The results of the KM (Kaplan–Meier estimator) curves revealed significant differences between the low- and high-risk groups (p=4.0e-5, **Figure 5D**). As shown in (**Figure 5E**), the TCGA-CESE data of the individual patients was assigned to low RiskScore or high RiskScore groups using the median RiskScore. The survival probability of patients with an elevated expression of IKZF3, APOBEC3H, JAK3, CLECL1, FOXP3, CD6, CLEC2D, LINC00158, and PILRA was increased. In addition, the application of receiver operating characteristic (ROC) curves verified the sensitivity and specificity of the overall survival model (**Figure 5F**). For the 1-, 3-, and 5-year prognoses, the present risk model predicted AUC values of 0.74, 0.68, and 0.71, respectively. This supported the accurate prediction of CESC patients’ overall survival by the model.

**Figure 6:**
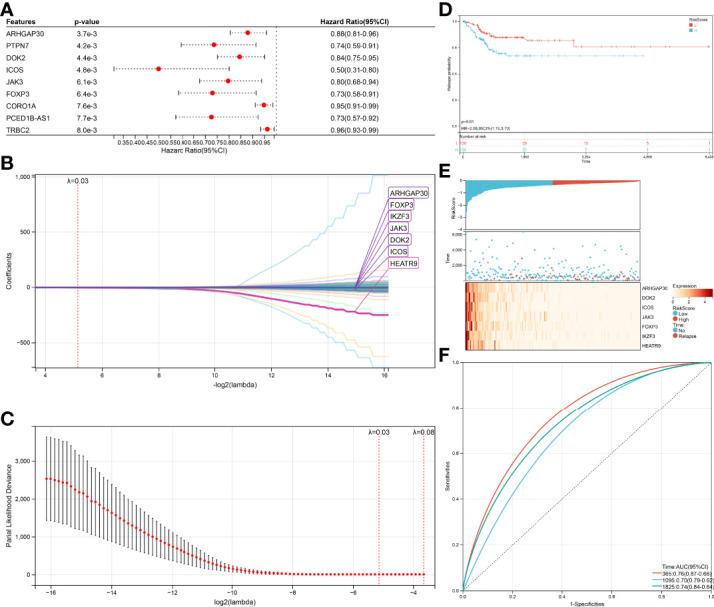
Verification of the prognostic risk models. **(A)** KM curves of different RiskScore in the survival-related model. **(B)** RiskScore and survival time with survival status profile and expression levels of the nine gene signatures. **(C)** ROC curve validation for survival-related model. **(D)** KM curves of different RiskScore in the relapse-related model. **(E)** RiskScore and relapse time with relapse status profile and expression levels of the seven gene signatures. **(F)** ROC curve validation for relapse-related model.

The same method was performed again to get a relapse model. One hundred thirty-nine genes were significantly associated (log-rank test p<0.05, **Figure 6A**) with relapse, 54 genes increased in hazard (HR>1) while 85 genes reduced in hazard (HR<1). The relapse model is optimal when lambda = 0.0283647620242562, seven genes (ARHGAP30, DOK2, ICOS, JAK3, FOXP3, IKZF3, HEATR9) were chosen to construct a relapse risk model (**Figures 6B, C**). The final 7-gene signature is as follows:

*RiskScore* = –0.0210123392211442 ∗ *ARHGAP*30 – 0.0139776933948472 ∗ *DOK*2 – 0.0654058831289185 ∗ *ICOS* – 0.00439045899467425 ∗ *JAK*3 – 0.0303215128344747 ∗ *FOXP*3 – 0.0203955365226131 ∗ *IKZF*3 – 1.57542330970538 ∗ *HEATR*9

An equal approach was used for the 7-gene relapse model utilizing the same models as for the survival model. A significantly higher relapse probability of the high RiskScore group was observed than in the low RiskScore group (**Figure 6D**, p=0.01). The relapse probability of patients with a reduced expression of ARHGAP30, DOK2, ICOS, JAK3, FOXP3, IKZF3, and HEATR9 was increased identifying the down-regulation of these genes as potential risk factor of relapse (**Figure 6E**). AUC values of 0.76 (1-year relapse), 0.70 (3-year relapse), and 0.74 (5-year relapse) obtained from the ROC curves support the high accuracy of the model in predicting CESC patients’ relapse (**Figure 6F**).

### Gene Set Enrichment Analysis

**Figure 7:**
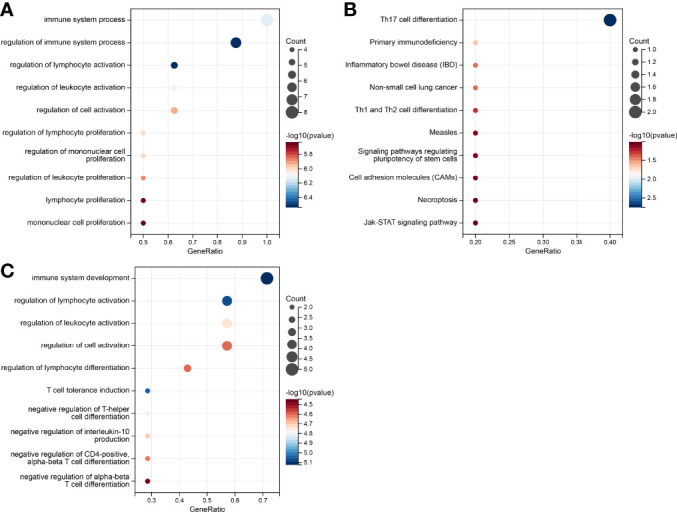
Gene set enrichment analysis of the genes in the prognostic risk models. **(A)** GO enrichment in the survival-related model. **(B)** KEGG pathways in survival-related model. **(C)** GO enrichment in the relapse-related model.

To access the non-immunological function of the hub genes, which showed highest correlation to the CD8+ T cells or B cells according to WGCNA analysis, GO functional and KEGG pathway analysis were performed. The outcome was that genes relevant in the survival risk model and relapse risk model were predominantly enriched in biological processes associated with immunological function, while the influence on non-immunological processes could be neglected (**Figures 7A–C**). Due to the small number of genes in the relapse risk model, no hits in the KEGG pathway analysis were obtained.

### HPV-Associated Pan Cancer Analysis

**Figure 8:**
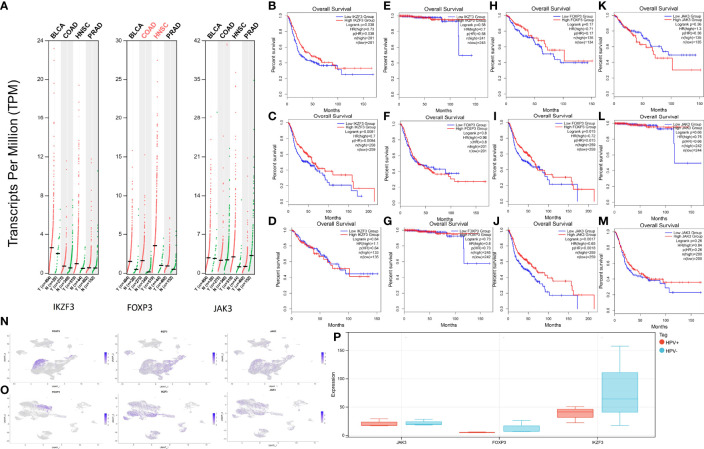
Expression levels and survival analysis in HPV-associated cancers. **(A)** IKZF3, FOXP3, and JAK3 expression levels in BLCA, HNSC, PRAD, and COAD. **(B)** Survival analysis between high-IKZF3 and low-IKZF3 groups in BLCA. **(C)** Survival analysis between high-IKZF3 and low-IKZF3 groups in HNSC. **(D)** Survival analysis between high-IKZF3 and low-IKZF3 groups in COAD. **(E)** Survival analysis between high-IKZF3 and low-IKZF3 groups in PRAD. **(F)** Survival analysis between high-FOXP3 and low-FOXP3 groups in BLCA. **(G)** Survival analysis between high-FOXP3 and low-FOXP3 groups in PRAD. **(H)** Survival analysis between high-FOXP3 and low-FOXP3 groups in COAD. **(I)** Survival analysis between high-FOXP3 and low-FOXP3 groups in HNSC. **(J)** Survival analysis between high-JAK3 and low-JAK3 groups in HNSC. **(K)** Survival analysis between high-JAK3 and low-JAK3 groups in COAD. **(L)** Survival analysis between high-JAK3 and low-JAK3 groups in PRAD. **(M)** Survival analysis between high-JAK3 and low-JAK3 groups in BLCA. **(N)** Expressions of FOXP3, IKZF3, JAK3 in scRNA-seq HPV- HNSC. **(O)** Expressions of FOXP3, IKZF3, JAK3 expression in scRNA-seq HPV+ HNSC. **(P)** Quantification of FOXP3, IKZF3, JAK3 in bulk RNA-seq HPV+ and HPV- HNSC.

Since HPV infection is suspected to contribute to the emergence of other cancer types (Bladder Urothelial Carcinoma (BLCA), HNSC, Colon adenocarcinoma (COAD), Prostate Adenocarcinoma (PRAD)), an HPV-associated pan cancer analysis of IKZF3, FOXP3, and JAK3 was performed. Since these genes were represented in both risk models (survival and relapse), their potential impact on cancerogenesis was estimated higher than for genes appearing in only one model. The expressions of genes are shown in **Figure 8A**.

The survival analysis of IKZF3 showed that IKZF3 is favorable in BLCA (**Figure 8B**) and HNSC (**Figure 8C**). No influence of IKZF3 on the survival probability was observed in COAD (**Figure 8D**) and PRAD (**Figure 8E**).

Differential expressions of FOXP3 showed no significant impact on BLCA (**Figure 8F**), PRAD (**Figure 8G**), and COAD (**Figure 8H**), while high expression of FOXP3 was associated with a higher percentage of survival in HNSC (**Figure 8I**).

HNSC patients with high expression of JAK3 tend to have more prolonged survival (**Figure 8J**), and there is no significant impact of JAK3 expressions on COAD (**Figure 8K**), PRAD (**Figure 8L**), and BLCA (**Figure 8M**).

In the scRNA analysis of HPV+ and HPV- HNSCC, the hub genes show a similar tendency as they do in HPV+ and HPV- CESC (**Figures 8N, O**). An intuitive difference could be overserved *via* the quantification of expression profiling by high throughput sequencing (**Figure 8P**).

## Discussion

Although it has been widely confirmed that HPV-associated lesions pose a higher risk to become malignant (4), there are few studies comparing immune cell infiltration, gene expression, and tumor heterogeneity between HPV+ and HPV- tumors. The current research shows that single-cell transcriptomics are a powerful tool to explore heterogeneity of HPV+ and HPV- tumors, which facilitates assessing the impact of HPV infection on cancer biology by identifying gene markers for diagnosis, prognosis, and therapy.

We assume that HPV affects the host from transcriptional state to immune infiltration. Therefore, we analyzed the transcriptional differences between HPV+ and HPV- samples by scRNA-seq as well as bulk RNA sequencing. Based on the differences in gene expression, we found that HPV+ samples showed CD8+ T cells and B cells were down-regulated while T reg cells, CD4+ T cells and epithelial cells were up-regulated in HPV+ CESC patients. The analysis of scRNA-seq is consistent with a previous study about immunologic treatments for CESC (17), HPV clearance in basal epithelial cells requires the activation of adaptive immunity and the formation of helper T cells that enable the development of CD8+ cytotoxic T cells against viral early proteins. In addition, T helper cells also support optimal activation of B cells, which can protect against subsequent infections at mucosal and systemic levels.

After learning that there was an immune infiltration difference between HPV+ and HPV- samples, we performed tumor immune infiltration of bulk RNA sequencing samples with survival information by CIBERSORT and found significantly longer survival for samples infiltrated with CD8+ cells and B cells. Therefore, WGCNA analysis was used to screen for differential genes (including low immune cell specificity genes) with the highest correlation to CD8+ cells and B cells and used for subsequent construction of survival and relapse models. Finally, we identified JAK3, FOXP3, and IKZF3, three genes with reduced expression in the HPV+ group, as protective factors for survival and relapse. The lower expression of these three, the higher the RiskScore in the model, which means a higher risk of death and recurrence.

The higher expression of three hub genes shows a higher percentage of survival in HNSC, which is consistent with our analyzed difference between HPV+ and HPV- HNSC samples. However, this result could not be observed in other potential HPV-associated cancers, only IKZF3 was found to be favorable in BLCA. As Jørgensen KR and Jensen JB found out, most studies fail to prove clear-cut relevance of HPV in BLCA irrespectively of histological subtype (18). No hub genes could associate expression level with survival state in COAD and PRAD. As KHOURY et al. mentioned (19), no HPV transcripts were detected in prostate and colon adenocarcinomas *via* RNA-Seq data. For this result, we concluded that hub genes are affected by HPV+ and that high expression of hub genes in non-HPV-infected tumors is not protective.

The functions of FOXP3, JAK3, and IKZF3 are widely researched in the field of tumor immunology. As the most specific and reliable biomarker of T reg cells, the studies of FOXP3 are conflicted. The infiltration of tumors with FOXP3+ Tregs was considered unfavorable for patient survival in many types of cancer. However, more recent work has described Helios as a new marker of Tregs. Tregs only have an immunosuppressive function when both FOXP3 and Helios are expressed (20). In the study of HPV+ tonsillar squamous cell carcinoma (20, 21), FOXP3 is a favorable prognostic factor. In addition, The Foxp3 gene was recognized as an immunological regulator, suppressing oncogenes while activating tumor suppressor genes (22). Decreased FOXP3 expression would impair FOXP3-mediated oncogene repression and tumor suppressor activation (23, 24). JAK3 is a tyrosine kinase that belongs to the Janus family of kinases, most commonly expressed in T cells and NK cells (25). Laffort et al. found that patients with HPV disease had severe combined immune deficiency associated with JAK3 deficiency. In addition, JAK3 mutations were identified in HPV+ Squamous Cell Carcinomas of the Oropharynx (OPSCC) (26) and Cervical Precancers (27). The production of IKZF3 is a transcription factor that is important in regulating B lymphocyte proliferation and differentiation and has been described as a target of HPV integration (28). In the study of OPSCC, IKZF3 RNA was detected in HPV+ cell lines, and regardless of tumor cell expression pattern, the immune cell infiltration was significantly positive for IKZF3. In this study, IKZF3 had a HR of 0.38, which indicated IKZF3 is protective (29). For the first time, we proposed two signatures based on the heterogeneity of CESC, which may be applied for survival and relapse, and provided potential immunotherapy targets. However, we did not explore the mechanism of genes in two signatures. Future efforts should focus on using many samples to verify the model’s accuracy and explore the molecular mechanism of the signature genes, providing experimental evidence for application to risk prediction and treatment in HPV- associated Cancers.

In conclusion, analysis of scRNA-seq and bulk RNA-seq of HPV+ and HPV- CESC samples revealed heterogeneity from transcriptional state to immune infiltration. Based on the difference, we found prognosis-related genes where expression was affected by HPV+. The LASSO Cox regression analysis of these genes constructed survival and recurrence models. IKZF3, FOXP3, and JAK3 represented in both risk models had the similar distribution and protective effects in HPV-associated HNSC, which means the expression of the hub genes are affected by HPV+.

## Data Availability Statement

Publicly available datasets were analyzed in this study. This data can be found here: https://www.ncbi.nlm.nih.gov/geo/query/acc.cgi?acc=GSE171894, PRJNA721342, https://www.ncbi.nlm.nih.gov/geo/query/acc.cgi?acc=GSE139324, https://www.ncbi.nlm.nih.gov/geo/query/acc.cgi?acc=GSE142583, https://www.ncbi.nlm.nih.gov/geo/query/acc.cgi?acc=GSE6791, https://www.ncbi.nlm.nih.gov/geo/query/acc.cgi?acc=GSE181805, https://www.ncbi.nlm.nih.gov/geo/query/acc.cgi?acc=GSE190224. In addtion, GSE139324, GSE 6791, GSE190224.

## Author Contributions

BC-E and MR conceived and designed the study. EW, AR, and JL analyzed the data and drafted the manuscript. EW and LF prepared the figures. All authors contributed to the article and approved the submitted version.

## Conflict of Interest

The authors declare that the research was conducted in the absence of any commercial or financial relationships that could be construed as a potential conflict of interest.

## Publisher’s Note

All claims expressed in this article are solely those of the authors and do not necessarily represent those of their affiliated organizations, or those of the publisher, the editors and the reviewers. Any product that may be evaluated in this article, or claim that may be made by its manufacturer, is not guaranteed or endorsed by the publisher.
