# Supplementary material for: Integration of scRNA-Seq and TCGA RNA-Seq to Analyze the Heterogeneity of HPV+ and HPV- Cervical Cancer Immune Cells and Establish Molecular Risk Models
Source: Front Oncol. 2022 Jun 1;12:860900. doi: 10.3389/fonc.2022.860900 (PMC9198569; doi:10.3389/fonc.2022.860900)
Supplement: Supplementary file 1 [file DataSheet_1.docx]

Supplementary Material

## Supplementary Figures


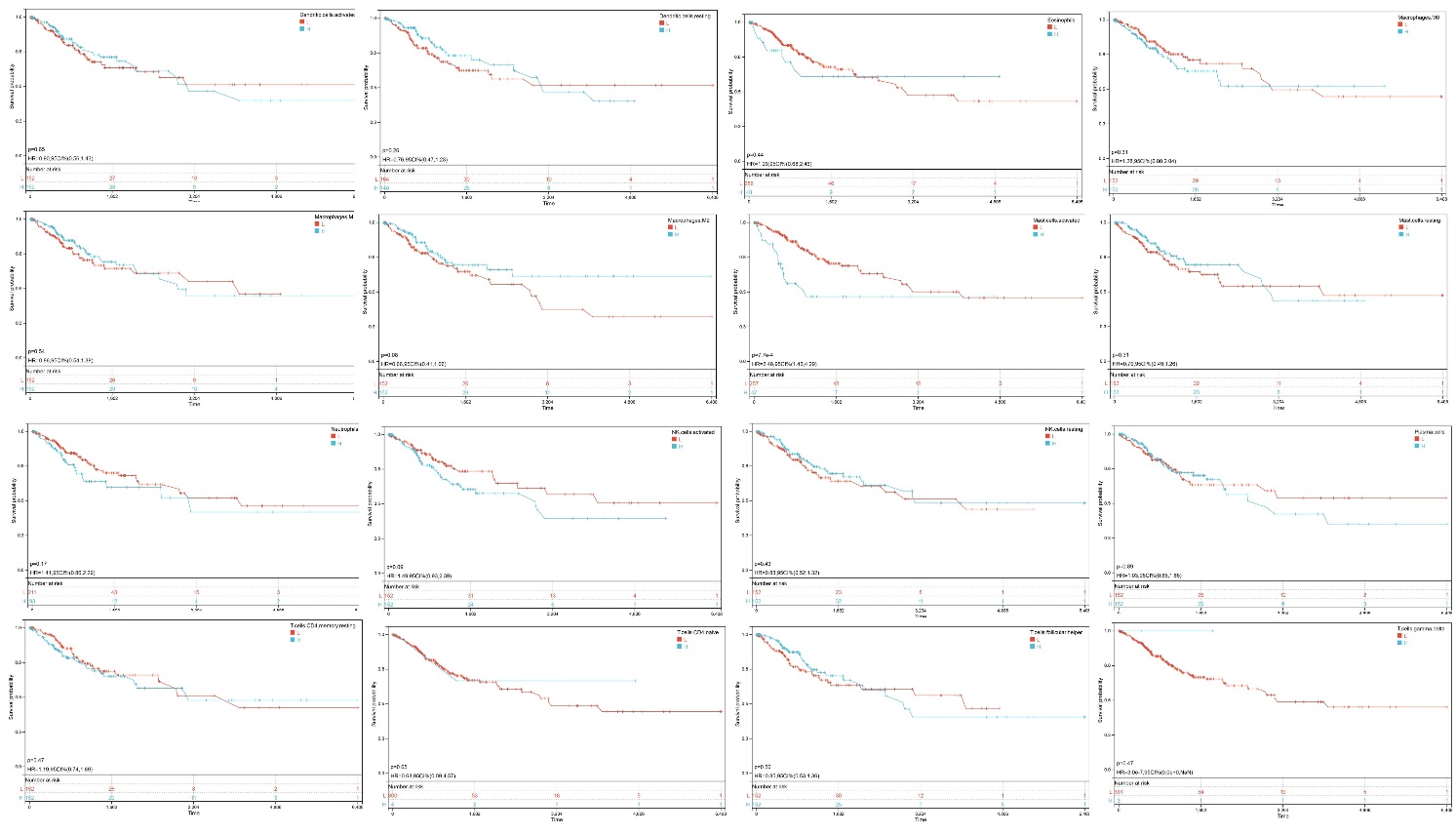


**Supplementary Figure 1.** KM curves of different expression immune cell types.

Different expression of other immune cell types showed no statistical influence on the survival probability of CESC patients.
